# Uniformity of HfO_2_ Thin Films Prepared on Trench Structures via Plasma-Enhanced Atomic Layer Deposition

**DOI:** 10.3390/nano13010161

**Published:** 2022-12-29

**Authors:** Boyun Choi, Hyeong-U Kim, Nari Jeon

**Affiliations:** 1 Department of Materials Science and Engineering, Chungnam National University, Daejeon 34134, Republic of Korea; 2 Department of Plasma Engineering, Korea Institute of Machinery & Materials (KIMM), Daejeon 34103, Republic of Korea

**Keywords:** plasma-enhanced atomic layer deposition, hafnium oxides, uniformity of thin films, wafer-scale uniformity, high aspect ratio, trench structures

## Abstract

In this study, we assessed the physical and chemical properties of HfO_2_ thin films deposited by plasma-enhanced atomic layer deposition (PEALD). We confirmed the self-limiting nature of the surface reactions involved in the HfO_2_ thin film’s growth by tracing the changes in the growth rate and refractive index with respect to the different dose times of the Hf precursor and O_2_ plasma. The PEALD conditions were optimized with consideration of the lowest surface roughness of the films, which was measured by atomic force microscopy (AFM). High-resolution X-ray photoelectron spectroscopy (XPS) was utilized to characterize the chemical compositions, and the local chemical environments of the HfO_2_ thin films were characterized based on their surface roughness and chemical compositions. The surface roughness and chemical bonding states were significantly influenced by the flow rate and plasma power of the O_2_ plasma. We also examined the uniformity of the films on an 8″ Si wafer and analyzed the step coverage on a trench structure of 1:13 aspect ratio. In addition, the crystallinity and crystalline phases of the thin films prepared under different annealing conditions and underlying layers were analyzed.

## 1. Introduction

Research on the deposition and characterization of HfO_2_ thin films has gained significant attention in the past 20 years because they are one of the major candidate materials for high-K dielectrics [1,2,3,4]. Related research has rapidly increased since the early 2000s, when intriguing ferroelectric properties were observed in HfO_2_ thin films with a thickness of a few nanometers [5,6,7]. Atomic layer deposition (ALD) and plasma-enhanced ALD (PEALD) have been largely employed for the deposition of HfO_2_ thin films, because ALD-based techniques have unique advantages, such as control over the film thickness at an Angstrom level and the high conformality of the film on the surfaces of complicated structures [8,9]. PEALD can offer additional advantages compared to thermal ALD in terms of an increased growth rate, decreased deposition temperature, and greater degree of freedom in the selection of ALD precursors [10,11]. Most previous HfO_2_ studies related to ALD and PEALD have focused on the optimization of the processing parameters, such as the deposition conditions, annealing conditions, and doping, to enhance the ferroelectric properties, such as the remnant polarization values and cycling stability [12,13,14]. In addition, DFT-based theoretical studies have been performed to understand the interplay between growth techniques and properties in HfO_2_ [15,16,17] and similar metal oxide systems [18,19]. Previously, the wafer-scale growth of thin films for various materials has been demonstrated via different growth techniques (e.g., ALD, molecular beam epitaxy, sputtering, and vapor transfer) [20,21,22,23,24]. However, only a few recent studies have focused on the large-scale uniformity and conformality of HfO_2_ films prepared by PEALD [25]. Our work shows the importance of the optimization of the PEALD conditions, not only for the film’s uniformity at a large wafer scale but also for the conformality at a nanometer scale. The experimental data set presented in this work can be well correlated with the simulations dealing with precursor flows both on a reactor scale and nanometer scale within the trench structures. Application fields of the ferroelectric HfO_2_ thin films include ferroelectric random-access memory and a ferroelectric field-effect transistor [26,27,28]. Surface roughness, film uniformity, and dielectric constants are important factors that determine the quality of thin films, which ultimately governs the properties of devices with thin films, such as leakage currents, capacitance, and operating voltage. Therefore, the smooth surface and good thickness uniformity of the HfO_2_ thin film can help to improve the performance aspects of the device, such as leakage current and capacitance; it is thus necessary to achieve a smooth surface and good conformality in the HfO_2_ thin film [29,30].

The main purpose of this work was to evaluate the uniformity and conformality of the HfO_2_ thin film deposited on a trench structure of an 8’’ wafer through PEALD. We characterized the HfO_2_ thin films deposited by PEALD in terms of growth per cycle (GPC), refractive index, surface roughness, average chemical composition, and local chemical environment, which were influenced by the O_2_ plasma conditions. Additionally, the effects of the annealing conditions and types of underlying layers on the crystallinity and crystalline phase of the thin films were studied.

## 2. Materials and Methods

Si wafers of 8’’ diameter (*p*-type, 1–10 Ωcm) were washed via sonication, by immersing them in ethanol for 15 min and then in isopropyl alcohol for 15 min. To prepare the TiN-coated samples, a ~100-nm-thick TiN layer was deposited on the Si wafer by sputtering (ENDURA-550, AMAT Inc., Santa Clara, CA, USA). ITO/glass (ITO glass, 10 Ω/sq, 1.1 T, OMNISCIENCE, Yongin-si, Gyeonggi-do, Korea) substrates were used after undergoing the same cleaning step. PEALD was performed using a custom-built reactor in a cross-flow system that could employ wafers up to 8″ in size. The reactor was equipped with a vacuum pump (W2V80, WSA Co., Ltd., Gunpo-si, Gyeonggi-do, Korea) at an ultimate pressure of ~50 mTorr. Inductively coupled remote plasma was employed in the system, with a plasma showerhead located 89 mm away from the sample plate. The plasma sensor probe (Wise Probe, P&A Solutions, Seoul, Korea) was located immediately below the plasma showerhead, with the probe positioned at the center of the chamber. Tetrakis(dimethylamido)hafnium(IV) (TDMAHf, EG Chem Co., Ltd., Gongju-si, Chungcheongnam-do, Korea) was utilized as the Hf precursor, and its canister was maintained at 70–80 °C. The growth temperature (substrate temperature) was maintained at 250 °C. The temperatures of the gas lines, gas shower head, and pumping lines were maintained at 150 °C. Ar flow as the carrier gas was maintained at 10, 20, and 50 sccm and at 15 and 50 sccm along the direction of the Hf precursor flow (cross-flow direction) and along the direction of O_2_ plasma (vertical flow through the plasma gas shower). A schematic of the reactor chamber is provided in the Supplementary Materials (Figure S1). The dose times of the TDMAHf and O_2_ plasma were the primary variables, and the purge times for both TDMAHf and O_2_ plasma were maintained at 90 s. The purge times were selected such that the chamber pressure returned to the base pressure within the stipulated times (Figure S2). A custom-built rapid thermal processor (JMON. Co., Daejeon, Korea) was utilized to anneal the thin films at different temperatures (500 and 600 °C) for 30 s, with a temperature ramp-up rate of 100 °C/min and constant N_2_ flow of 50 sccm.

The thickness and refractive index of the deposited thin film were characterized using a spectroscopic ellipsometer (M-2000, J. A. Wollam Co., Lincoln, NE, USA) in the wavelength range of 370.74 –998.89 nm at reflection angles of 65°, 70°, and 75°. The uniformity of the 8″ wafer scale was measured at intervals of 2.0 cm. The SE data were analyzed using the Cauchy model, where the extinction coefficient is assumed to be zero. An example of SE analysis is given in Supplementary Materials (Figure S3). X-ray photoelectron spectroscopy (K-alpha, Thermo Fisher Scientific Inc., Waltham, MA, USA) was performed with an Al K-alpha source of 1 keV after surface etching by Ar^+^ ions for 5 s. Cross-sectional scanning electron microscopy (Verios 5 UC, Thermo Fisher Scientific Inc., Waltham, MA, USA) was performed on the trench structures of 1:13 aspect ratio. Grazing-incidence angle X-ray diffraction (GIXRD) was performed (D8 DISCOVER, Bruker AXS, GmbH, Karlsruhe, Germany) with a two-theta scan mode at a scan speed of 5 s/step and scan step of 0.03°.

## 3. Results

**Figure 1:**
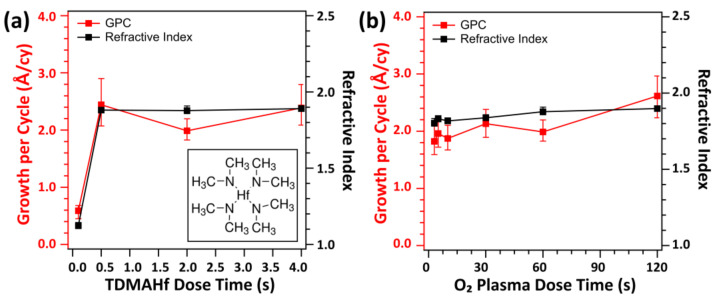
GPC and refractive index of HfO_2_ films deposited at different conditions of (**a**) TDMAHf (inset: molecular structure of TDMAHf) and (**b**) O_2_ plasma doses. For the experiment shown in (**a**), O_2_ plasma condition was maintained as follows: O_2_ gas flow of 50 sccm and O_2_ plasma power of 20 W. For the experiment shown in (**b**), TDMAHf dose time was kept at 2 s.

Figure 1 shows the growth per cycle (GPC) and refractive index (n) of the deposited HfO_2_ thin film as a function of the TDMAHf dose time (0.1, 0.5, 2, and 4 s) and O_2_ plasma dose time (3, 5, 10, 30, 60, and 120 s). In each growth condition, the number of ALD cycles was maintained at 100. Three samples were measured at the middle position of the precursor inlet and outlet. The measured GPC value appeared to be saturated to ~2.4 Å/cy at a ~0.5 s TDMAHf dose under an O_2_ plasma dose time of 60 s. The GPC value in this study was similar to or slightly greater than those reported by other groups [11,25,31,32]. The refractive index was well saturated to ~1.9 at the same TDMAHf dose time of 2 s, which is similar to the values obtained in well-densified HfO_2_ thin films [25,31]. We also varied the dose time of O_2_ plasma with that of TDMAHf maintained at 2 s. While the n values were almost constant regardless of the plasma time, GPC tended to gradually increase from 1.9 Å/cy to 2.4 Å/cy with an increase in the O_2_ plasma time. The gradual increase in GPC with increasing O_2_ plasma time can be related to the surface roughening and larger surface density of reactive sites induced by prolonged plasma exposure [33,34,35].

**Figure 2:**
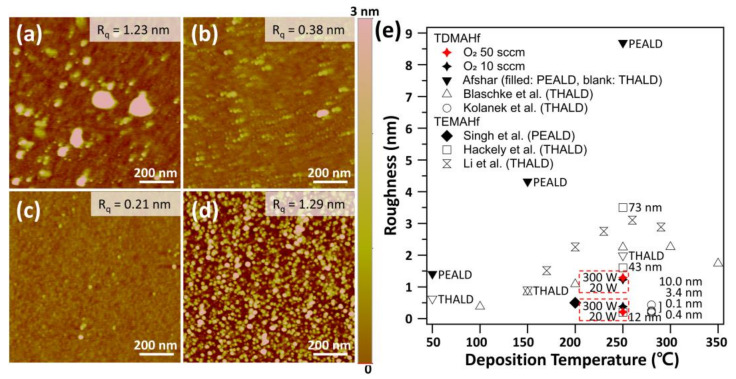
Surface roughness of the HfO_2_ films deposited at different flow rates and plasma power of O_2_: (**a**) 10 sccm and 20 W, (**b**) 10 sccm and 300 W, (**c**) 50 sccm and 20 W, and (**d**) 50 sccm and 300 W. (**e**) Comparisons of surface roughness of HfO_2_ films in our work and those reported by other research groups [9,36,37,38,39,40].

We further optimized the PEALD conditions in terms of the O_2_ flow rate and plasma power, while keeping the dose times for TDMAHf and O_2_ plasma constant (TDMAHf:2 s, O_2_ plasma:60 s). Figure 2a shows the atomic force microscopy (AFM) images of the four samples prepared under different O_2_ flow (10 sccm vs. 50 sccm) and plasma power (20 W, 300 W) conditions. During these experiments, the flow rate of the Ar carrier gas was kept constant; therefore, the chamber pressure was varied according to the variation in the O_2_ flow rate. For all samples, the surface roughness was in the range of a few Å to a few nm depending on the experimental conditions. To obtain smoother films, a low O_2_ flow rate (10 sccm) with high plasma power (300 W) or a high O_2_ flow rate (50 sccm) with low plasma power (20 W) was preferred. The optimized surface roughness values obtained in our work were considerably lower than those reported in the literature, including both the thermal ALD and PEALD processes with the same Hf precursor. Table 1 summarizes the ion density, ion flux, and electron temperature as a function of the O_2_ flow rate and plasma power. All three parameters were sensitive to changes in the plasma power rather than the O_2_ flow rate.

**Figure 3:**
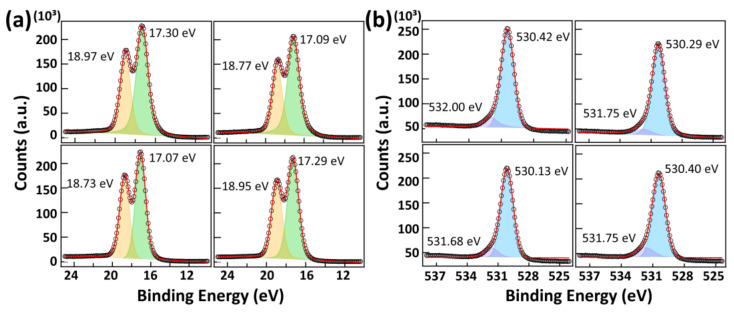
HRXPS spectra of the HfO_2_ films deposited at different O_2_ flow rates and O_2_ plasma power: (**a**) the Hf 4f XPS spectra, (**b**) the O 1s XPS spectra of the as-deposited HfO_2_ films. (Top panel: O_2_ flow rate 10 sccm, lower panel: O_2_ flow rate 50 sccm, left side panel: O_2_ plasma power 20 W, right side panel: O_2_ plasma power 300 W).

The chemical composition and local chemical environment of each constituent element of the four films were characterized using X-ray photoelectron spectroscopy. As shown in Table 2, all four samples were slightly rich in oxygen (Hf:O ≈ 1:2.4), with an N impurity concentration of ~5 at. %. The O-rich nature may have arisen from the native SiO_2_ layer of ~2 nm because the thickness of the film was ~13 nm. The samples prepared with low O_2_ flow had a lower C impurity concentration than those prepared with high O_2_ flow. The detected C and N impurity levels are similar to those reported in the literature [9,25,41,42]. Figure 3 shows the Hf 4f HRXPS and O 1s HRXPS spectra of the four different films. Hf 4f HRXPS are well deconvoluted into two peaks of Hf 4f 5/2 and Hf 4f 7/2 of symmetrical shape, located at ~18.8 eV and ~17.2 eV, respectively. The Hf 4f HRXPS spectrum could be deconvoluted into four peaks; however, in the four-peak analysis, the BEs of the two adjacent peaks were too close (0.1~0.5 eV), as shown in Figure S4. The O 1s HRXPS spectra were deconvoluted into two peaks, Hf-O (~530.3 eV) and Hf-OH (~531.7 eV). A higher BE peak may also include the contribution of oxygen vacancies. However, PEALD processes are known to produce HfO_2_ thin films with a lower concentration of oxygen vacancies compared to thermal ALD processes [43]. The Hf-OH components are commonly found in HfO_2_ thin films deposited by both ALD and PEALD [9,44,45]. The ratio of the Hf-O component to the Hf–OH component is sensitive to the oxygen flow rate. The relative contribution of the Hf-O component can be improved by increasing the oxygen flow. This trend is observed because a higher O_2_ flow rate increases the partial pressure of oxygen. The relative contributions of the Hf-OH vs. Hf-O components are less sensitive to the plasma power.

**Figure 4:**
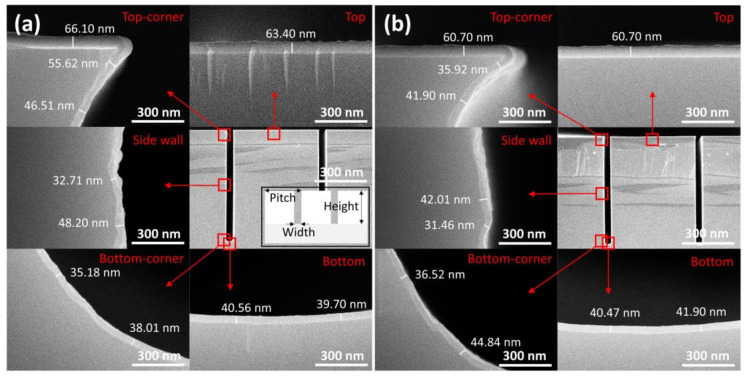
UHR FE-SEM images of (**a**) as-deposited and (**b**) annealed HfO_2_ films at different locations in the trench. The locations where higher-magnification SEM images were captured are highlighted by red boxes and arrows. The inset of (**a**) shows the schematic of a trench patterned wafer. (Trench width: 5.0 μm, height: 65.0 μm, pitch: 52.5 μm).

The film conformality was tested on a patterned wafer containing trench structures with an aspect ratio of 1:13. For the preparation of this sample, the optimized PEALD conditions (TDMAHf dose: 2 s, O_2_ plasma dose: 60 s, O_2_ flow: 50 sccm, and plasma power: 20 W) were used. As shown in Figure 4a, the film thickness varied depending on the location of the trench structures, which was likely related to the distribution of the reactive species in the plasma. The step coverage of the as-deposited film, calculated by comparing the thicknesses at the top and bottom surfaces, was ~64%. The film thickness on the sidewall was lower than that on the bottom surface, indicating the critical role of the isotropic nature of the reactive species in the plasma. Conformality was also measured after rapid thermal annealing (RTA). The film thickness was slightly decreased at all locations by ~4.3–8.2% due to film densification. After the RTA, the step coverage was measured to be ~69%. There was no correlation between the degree of densification and the location within the trench.

**Figure 5:**
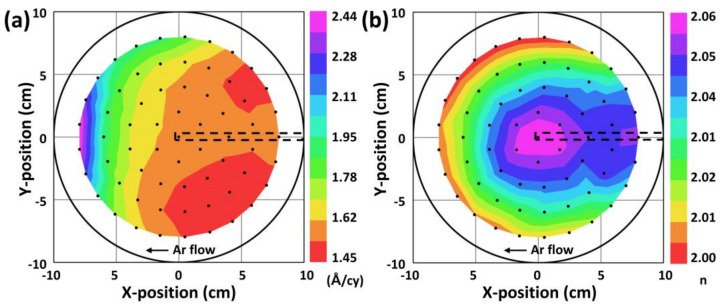
Uniformity of (**a**) GPC and (**b**) refractive index at the 8″ wafer-scale measured by SE. The dots represent the locations at which SE data were collected.

The film uniformity at the 8″ wafer scale was tested under the optimized PEALD conditions. As shown in Figure 5a, the GPC value tended to increase along the direction from the precursor inlet to the precursor outlet, with a large uniform area near the center and precursor inlet location. The non-perfect uniformity in GPC at a large scale is not likely related to the insufficient dosage of the TDMAHf precursor because well-saturated behavior was observed at the TDMAHf dose time of > 2 s. Although the GPC distribution resembled the direction of the TDMAHf precursor flow, we instead hypothesized that the non-uniformity may have arisen from the non-uniform distribution of plasma species. This is because the GPC value was not completely saturated as a function of the O_2_ plasma time (Figure 1b) in the ALD reactor. The GPC values measured at the 8″ wafer scale were lower than those measured from samples ~1 cm in size (Figure 1). The discrepancy in the GPC values may have arisen from differences in the size and number of samples used, as well as the type of sample platter. However, an 8″ Al sample platter was fully covered by the 8″ Si wafer in the wafer-scale experiment, and much of the Al platter surface was exposed in the GPC saturation experiment. Although the specific effects of the exposed Al surface of the sample platter could not be identified, it may have varied the distribution of reactive species in the plasma. Furthermore, the presence of the plasma sensor probe appeared to influence the plasma distribution because the GPC distribution resembled the location of the probe, as depicted by the black dashed line in Figure 5a. Despite the non-perfect uniformity of the film, the overall variation in the refractive index was insignificant, with a standard deviation of 0.02. Similar effects were also observed in the presence of the plasma probe. Moreover, as shown in Figure 5b, the refractive index may vary depending on the density of the thin films, the inclusion of functional groups such as –OH, and impurities. Future work will focus on evaluating the distribution of GPC and the refractive index with different positions of the plasma probe.

**Figure 6:**
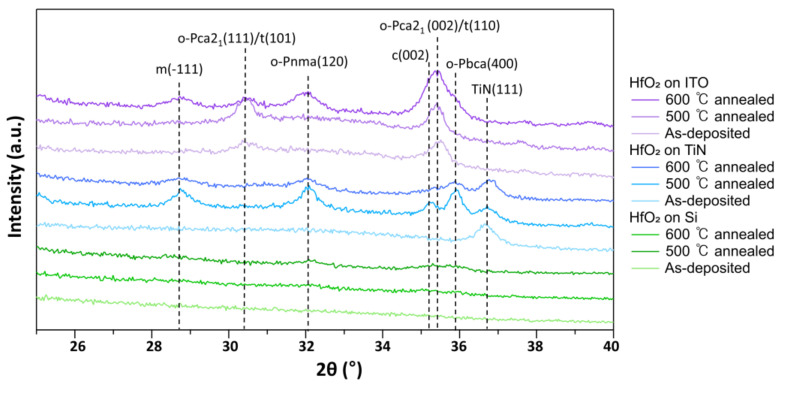
GIXRD of HfO_2_ film of as-deposited state and annealed state on Si, TiN, and ITO.

Finally, we evaluated the crystallinity of the PEALD HfO_2_ films deposited on different underlying layers (Si, TiN, and ITO) and under different RTA conditions (Figure 6). The film thicknesses of the tested samples were ~61 nm. The crystallinity of the film was enhanced with the TiN or ITO underlying layer, compared to a bare substrate, regardless of the RTA temperature. For the ITO samples, orthorhombic (o) and/or tetragonal (t) phases were identified by the peak at ~30.4° in the as-deposited states. The distinction between the o and t phases was not clear at this point because the two phases presented a peak at very similar locations: o (Pnma) at 30.3° (ICDD PDF card 01-087-2106), o (Pca21) at 30.4° [46], o (Pbca) at 30.4° (ICDD PDF card 01-081-0028), and t at 30.3° (ICDD PDF card 01-078-5756). The o/t phase was further enhanced by RTA at 500 °C, and the monoclinic (m) phase became more dominant than the other phases by RTA at 600 °C. A rather broad peak at ~32° in the ITO samples annealed at 600 °C may imply the presence of the o (Pnma) phase along with an m phase. In the set of ITO samples, a dominant peak at ~35.4° was consistently observed, mainly due to the o(002)/t(110) phase, although there could be a minor contribution of the cubic (c) phase of (002), with its peak expected to appear at ~35.2° (ICDD PDF card 00-053-0560). Interestingly, the TiN sample with 500 °C RTA exhibited better crystallinity than the sample with 600 °C RTA, with a small contribution from the cubic (c) phase at ~35.2°. It is interesting to note that the ITO sample had a higher portion of o/t phase compared to the TiN samples for the same RTA temperature of 500 °C. The crystallinity and crystalline phases of the HfO_2_ thin films evolved after RTA depend on the types of underlying layers (TiN, ITO, or SiO_2_), implying the presence of an interplay between the thermal stability/thermal stress of the as-grown HfO_2_ thin films and the RTA process [47,48,49,50]. The transition from the o/t phase to the m phase is correlated to the presence of a critical grain size above which the m phase becomes favorable [12]. Considering that the ITO and TiN samples had identical film thicknesses, it was possible that the underlying ITO layer tended to retard grain growth. Furthermore, previous works reported that HfO_2_ thin films, predominantly exhibiting o/t phases, had a thickness typically of the order of a few nanometers. This can also be understood in terms of the presence of a critical grain size because the average grain size tends to increase with the film thickness. In this context, our results showing the predominant o/t phase at a ~61 nm film thickness without any doping imply that the process range for obtaining the o/t phase can be further widened with the appropriate choice of the underlying layer.

## 4. Conclusions

In this work, we studied the physical and chemical properties of HfO_2_ films deposited by PEALD, with an emphasis on the plasma conditions. While GPC showed well-saturated behavior as a function of the TDMAHf dose time, it tended to gradually increase with the increasing O_2_ plasma time. The O_2_ flow rate and plasma power in the O_2_ plasma half-cycle were optimized to obtain smooth films with Å-level surface roughness. HRXPS analysis of the deposited films under different plasma conditions showed primarily Hf-O bonding signals, with a minor contribution of –OH components and C and N impurities below ~4 and ~6 at. %, respectively. The 8″-scale SE mapping showed that the GPC varied within ~±25%, whereas the variation in the refractive index was only ~3%. The developed PEALD process resulted in step coverage of ~69%, measured from trench structures with an aspect ratio of 1:13. Finally, the crystallinity of the HfO_2_ PEALD film showed a strong dependency on the RTA conditions and types of underlying layers.

## Figures and Tables

**Table 1:** Plasma properties measured at the center of the wafer.

	Ion Density (/cm^3^)	Ion Flux (mA/cm^2^)	Electron Temperature (eV)
O_2_ flow rate 10 sccm, O_2_ plasma power 20 W	3.28 × 10^8^	0.03749	2.3780
O_2_ flow rate 10 sccm, O_2_ plasma power 300 W	8.03 × 10^9^	0.40201	3.0978
O_2_ flow rate 50 sccm, O_2_ plasma power 20 W	1.47 × 10^8^	0.02445	2.4841
O_2_ flow rate 50 sccm, O_2_ plasma power 20 W	4.28 × 10^9^	0.25453	3.1347

**Table 2:** Atomic concentrations of Hf, O, C, and N in HfO_2_ films deposited at different O_2_ flow rates and O_2_ plasma power.

Conditions	Atomic Concentrations (at.%)				Atomic Ratio
	C 1s	N 1s	O 1s	Hf 4f	Hf:O
(a) 10 sccm and 20 W	2.53	4.86	65.53	27.08	1:2.42
(b) 10 sccm and 300 W	2.31	4.64	65.90	27.15	1:2.43
(c) 50 sccm and 20 W	4.25	5.75	63.38	26.62	1:2.38
(d) 50 sccm and 300 W	4.12	5.66	63.22	26.99	1:2.34

## Data Availability

The data presented in this study are available on request from the corresponding author.

## Supplementary Materials

The following supporting information can be downloaded at: https://www.mdpi.com/article/10.3390/nano13010161/s1, Figure S1: A schematic of the PEALD reactor chamber.; Figure S2: The pressure log of a supercycle consisting of TDMAHf half cycle (TDMAHf dose–purge) and O_2_ plasma half cycle (O_2_ plasma dose–purge).; Figure S3: An example of SE data analyzed using Cauchy equation.; Figure S4: The Hf 4f HRXPS spectra deconvoluted into four peaks of the HfO_2_ films deposited at different O_2_ plasma conditions. (Top panel: O_2_ flow rate 10 sccm, lower panel: O_2_ flow rate 50 sccm, left panel: O_2_ plasma power 20 W, right panel: O_2_ plasma power 300 W).

## References

[1] Cho Y.J., Nguyen N., Richter C., Ehrstein J., Lee B.H., Lee J.C. (2002). Spectroscopic ellipsometry characterization of high-k dielectric HfO_2_ thin films and the high-temperature annealing effects on their optical properties. Appl. Phys. Lett..

[2] Jones M., Kwon Y., Norton D. (2005). Dielectric constant and current transport for HfO_2_ thin films on ITO. Appl. Phys. A.

[3] Suzuki K., Kato K. (2009). Sol–gel synthesis of high-k HfO_2_ thin films. J. Am. Ceram. Soc..

[4] Tirmali P., Khairnar A.G., Joshi B.N., Mahajan A.M. (2011). Structural and electrical characteristics of RF-sputtered HfO_2_ high-k based MOS capacitors. Solid-State Electron..

[5] Polakowski P., Müller J. (2015). Ferroelectricity in undoped hafnium oxide. Appl. Phys. Lett..

[6] Sang X., Grimley E.D., Schenk T., Schroeder U., LeBeau J.M. (2015). On the structural origins of ferroelectricity in HfO_2_ thin films. Appl. Phys. Lett..

[7] Mueller S., Muller J., Schroeder U., Mikolajick T. (2012). Reliability Characteristics of Ferroelectric Si:HfO_2_ Thin Films for Memory Applications. IEEE Trans. Device Mater. Reliab..

[8] Choi S.-N., Moon S.-E., Yoon S.-M. (2019). Film thickness-dependent ferroelectric polarization switching dynamics of undoped HfO_2_ thin films prepared by atomic layer deposition. Ceram. Int..

[9] Hackley J.C., Gougousi T. (2009). Properties of atomic layer deposited HfO_2_ thin films. Thin Solid Films.

[10] Hur J., Tasneem N., Choe G., Wang P., Wang Z., Khan A.I., Yu S. (2020). Direct comparison of ferroelectric properties in Hf_0.5_Zr_0.5_O_2_ between thermal and plasma-enhanced atomic layer deposition. Nanotechnology.

[11] Nigro R.L., Schilirò E., Mannino G., Di Franco S., Roccaforte F. (2020). Comparison between thermal and plasma enhanced atomic layer deposition processes for the growth of HfO_2_ dielectric layers. J. Cryst. Growth.

[12] Kim K., Park M., Kim H., Kim Y., Moon T., Lee Y., Hyun S., Gwon T., Hwang C. (2016). Ferroelectricity in undoped-HfO_2_ thin films induced by deposition temperature control during atomic layer deposition. J. Mater. Chem. C.

[13] Lomenzo P.D., Takmeel Q., Moghaddam S., Nishida T. (2016). Annealing behavior of ferroelectric Si-doped HfO_2_ thin films. Thin Solid Films.

[14] Park M.H., Schenk T., Schroeder U., Uwe S., Cheol Seong H., Hiroshi F. (2019). Dopants in atomic layer deposited HfO_2_ thin films. Ferroelectricity in Doped Hafnium Oxide: Materials, Properties and Devices.

[15] Cortez-Valadez M., Fierro C., Farias-Mancilla J., Vargas-Ortiz A., Flores-Acosta M., Ramírez-Bon R., Enriquez-Carrejo J., Soubervielle-Montalvo C., Mani-Gonzalez P. (2016). Comparison of HfCl_4_, HfI_4_, TEMA-Hf, and TDMA-Hf as precursors in early growing stages of HfO_2_ films deposited by ALD: A DFT study. Chem. Phys..

[16] Huan T.D., Sharma V., Rossetti G.A., Ramprasad R. (2014). Pathways towards ferroelectricity in hafnia. Phys. Rev. B.

[17] Oh I.-K., Park B.-E., Seo S., Yeo B.C., Tanskanen J., Kim W.-H., Kim H. (2018). Comparative study of the growth characteristics and electrical properties of atomic-layer-deposited HfO_2_ films obtained from metal halide and amide precursors. J. Mater. Chem. C.

[18] Kakanakova-Georgieva A., Giannazzo F., Nicotra G., Cora I., Gueorguiev G.K., Persson P.O., Pécz B. (2021). Material proposal for 2D indium oxide. Appl. Surf. Sci..

[19] Dos Santos R.B., Rivelino R., Gueorguiev G.K., Kakanakova-Georgieva A. (2021). Exploring 2D structures of indium oxide of different stoichiometry. CrystEngComm.

[20] Pyeon J.J., Kim S.H., Jeong D.S., Baek S.-H., Kang C.-Y., Kim J.-S., Kim S.K. (2016). Wafer-scale growth of MoS_2_ thin films by atomic layer deposition. Nanoscale.

[21] Zhang H.-T., Zhang L., Mukherjee D., Zheng Y.-X., Haislmaier R.C., Alem N., Engel-Herbert R. (2015). Wafer-scale growth of VO_2_ thin films using a combinatorial approach. Nat. Commun..

[22] Kim H.-S., Patel M., Kim J., Jeong M.S. (2018). Growth of wafer-scale standing layers of WS_2_ for self-biased high-speed UV–visible–NIR optoelectronic devices. ACS Appl. Mater. Interfaces.

[23] Patel M., Kim H.-S., Kim J. (2017). Wafer-scale production of vertical SnS multilayers for high-performing photoelectric devices. Nanoscale.

[24] Patel M., Nguyen T.T., Kumar M., Ban D.-K., Won D., Zhao M., Kim J., Kim Y.K., Yang H., Wong C.-P. (2020). 2D layer-embedded transparent photovoltaics. Nano Energy.

[25] Lapteva M., Beladiya V., Riese S., Hanke P., Otto F., Fritz T., Schmitt P., Stenzel O., Tünnermann A., Szeghalmi A. (2021). Influence of temperature and plasma parameters on the properties of PEALD HfO_2_. Opt. Mater. Express.

[26] Schroeder U., Park M.H., Mikolajick T., Hwang C.S. (2022). The fundamentals and applications of ferroelectric HfO_2_. Nat. Rev. Mater..

[27] Park M.H., Lee Y.H., Mikolajick T., Schroeder U., Hwang C.S. (2018). Review and perspective on ferroelectric HfO_2_-based thin films for memory applications. Mrs Commun..

[28] Choi S.-N., Moon S.-E., Yoon S.-M. (2020). Impact of oxide gate electrode for ferroelectric field-effect transistors with metal-ferroelectric-metal-insulator-semiconductor gate stack using undoped HfO_2_ thin films prepared by atomic layer deposition. Nanotechnology.

[29] Zhang W., Zhou D., Sun N., Wang J., Li S. (2021). Effect of Bias Voltage on Substrate for the Structure and Electrical Properties of Y: HfO_2_ Thin Films Deposited by Reactive Magnetron Co-Sputtering. Adv. Electron. Mater..

[30] Cremers V., Puurunen R.L., Dendooven J. (2019). Conformality in atomic layer deposition: Current status overview of analysis and modelling. Appl. Phys. Rev..

[31] Melo L., Burton G., Kubik P., Wild P. (2016). Long period gratings coated with hafnium oxide by plasma-enhanced atomic layer deposition for refractive index measurements. Opt. Express.

[32] Provine J., Schindler P., Torgersen J., Kim H.J., Karnthaler H.-P., Prinz F.B. (2016). Atomic layer deposition by reaction of molecular oxygen with tetrakisdimethylamido-metal precursors. J. Vac. Sci. Technol. A Vac. Surf. Film..

[33] Alam A., Howlader M., Deen M. (2014). The effects of oxygen plasma and humidity on surface roughness, water contact angle and hardness of silicon, silicon dioxide and glass. J. Micromech. Microeng..

[34] Heo J.H., Ryu H., Lee W.-J. (2013). Effect of O_2_ plasma pretreatment on structural and optical properties of ZnO films on PES substrate by atomic layer deposition. J. Ind. Eng. Chem..

[35] Zhu Z., Sippola P., Lipsanen H., Savin H., Merdes S. (2018). Influence of plasma parameters on the properties of ultrathin Al_2_O_3_ films prepared by plasma enhanced atomic layer deposition below 100 °C for moisture barrier applications. Jpn. J. Appl. Phys..

[36] Afshar A. (2014). Materials Characterization and Growth Mechanisms of ZnO, ZrO_2_, and HfO_2_ Deposited by Atomic Layer Deposition. Ph.D. Thesis.

[37] Blaschke D., Munnik F., Grenzer J., Rebohle L., Schmidt H., Zahn P., Gemming S. (2020). A correlation study of layer growth rate, thickness uniformity, stoichiometry, and hydrogen impurity level in HfO_2_ thin films grown by ALD between 100 °C and 350 °C. Appl. Surf. Sci..

[38] Kolanek K., Tallarida M., Michling M., Schmeisser D. (2012). In situ study of the atomic layer deposition of HfO_2_ on Si. J. Vac. Sci. Technol. A Vac. Surf. Film..

[39] Singh R., Panigrahi J., Singh P. (2016). Plasma assisted atomic layer deposited hafnium oxide films for silicon surface passivation. RSC Adv..

[40] Li S., Zhang Y., Yang D., Yang W., Chen X., Zhao H., Hou J., Yang P. (2020). Structure and optical properties of HfO_2_ films on Si (100) substrates prepared by ALD at different temperatures. Phys. B Condens. Matter.

[41] Foroughi Abari A. (2012). Atomic Layer Deposition of Metal Oxide Thin Films on Metallic Substrates. Ph.D. Thesis.

[42] Kukli K., Pilvi T., Ritala M., Sajavaara T., Lu J., Leskelä M. (2005). Atomic layer deposition of hafnium dioxide thin films from hafnium tetrakis (dimethylamide) and water. Thin Solid Films.

[43] Martínez-Puente M., Horley P., Aguirre-Tostado F., López-Medina J., Borbón-Nuñez H., Tiznado H., Susarrey-Arce A., Martínez-Guerra E. (2022). ALD and PEALD deposition of HfO_2_ and its effects on the nature of oxygen vacancies. Mater. Sci. Eng. B.

[44] Triyoso D., Liu R., Roan D., Ramon M., Edwards N., Gregory R., Werho D., Kulik J., Tam G., Irwin E. (2004). Impact of deposition and annealing temperature on material and electrical characteristics of ALD HfO_2_. J. Electrochem. Soc..

[45] Zanders D., Ciftyurek E., Subaşı E., Huster N., Bock C., Kostka A., Rogalla D., Schierbaum K., Devi A. (2019). PEALD of HfO_2_ thin films: Precursor tuning and a new near-ambient-pressure XPS approach to in situ examination of thin-film surfaces exposed to reactive gases. ACS Appl. Mater. Interfaces.

[46] Miakonkikh A., Lomov A., Rogozhin A., Rudenko K., Lukichev V., Kiselev D., Tikhonenlo F., Antonov V., Popov V. Phase transformation in ALD hafnia based layers for silicon-on-ferroelectric devices. Proceedings of the 2020 Joint International EUROSOI Workshop and International Conference on Ultimate Integration on Silicon (EUROSOI-ULIS).

[47] Cho M.-H., Roh Y., Whang C., Jeong K., Nahm S., Ko D.-H., Lee J.H., Lee N., Fujihara K. (2002). Thermal stability and structural characteristics of HfO_2_ films on Si (100) grown by atomic-layer deposition. Appl. Phys. Lett..

[48] Kim S., Kim J., Choi J., Kang H., Jeon H., Bae C. (2006). Characteristics of HfO_2_ thin films deposited by plasma-enhanced atomic layer deposition using O_2_ plasma and N_2_O plasma. J. Vac. Sci. Technol. B Microelectron. Nanometer Struct. Process. Meas. Phenom..

[49] Nie X., Ma D., Ma F., Xu K. (2017). Thermal stability, structural and electrical characteristics of the modulated HfO_2_/Al_2_O_3_ films fabricated by atomic layer deposition. J. Mater. Sci..

[50] Wan J., Chen X., Ji L., Tu Z., Wu H., Liu C. (2022). Ferroelectricity of Hf_0.5_Zr_0.5_O_2_ Thin Films Free from the Influence of Electrodes by Using Al_2_O_3_ Capping Layers. IEEE Trans. Electron Devices.

