# Supplementary material for: Uniformity of HfO2 Thin Films Prepared on Trench Structures via Plasma-Enhanced Atomic Layer Deposition
Source: Nanomaterials (Basel). 2022 Dec 29;13(1):161. doi: 10.3390/nano13010161 (PMC9823614; doi:10.3390/nano13010161)
Supplement: Supplementary file 1 [file nanomaterials-13-00161-s001.zip › nanomaterials-2105978-supplementary.pdf]

Supplementary Materials:

Uniformity of HfO_2 Thin Films Prepared on Trench Structures via Plasma-Enhanced Atomic Layer Deposition

Boyun Choi [1], Hyeong-U Kim [2] and Nari Jeon [1,*]

[1] Department of Materials Science and Engineering, Chungnam National University, Daejeon 34134, Republic of Korea
[2] Department of Plasma Engineering, Korea Institute of Machinery & Materials (KIMM), Daejeon 34103, Republic of Korea
* Correspondence: njeon@cnu.ac.kr

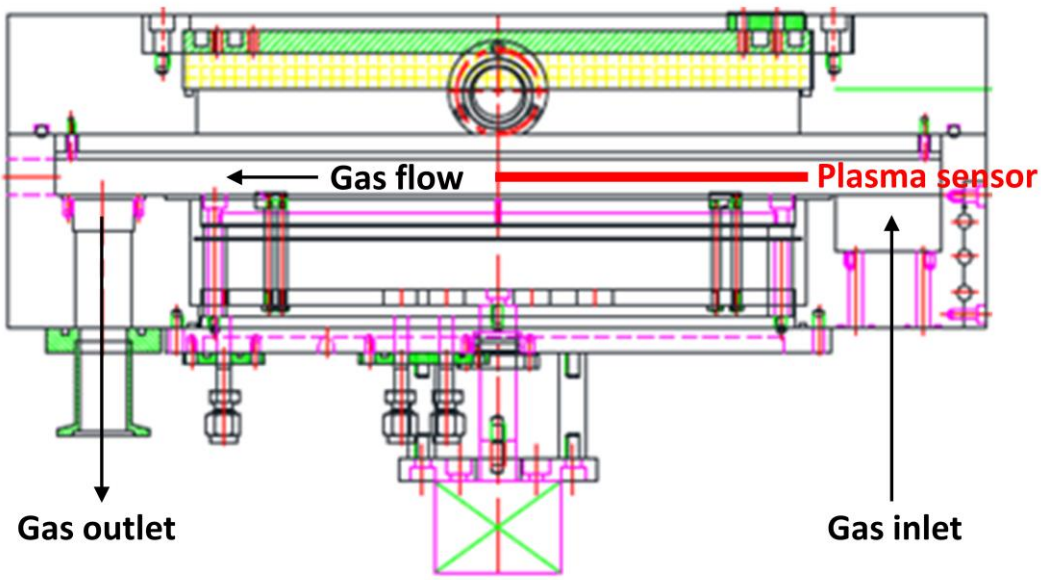

Figure S1. A schematic of the PEALD reactor chamber.

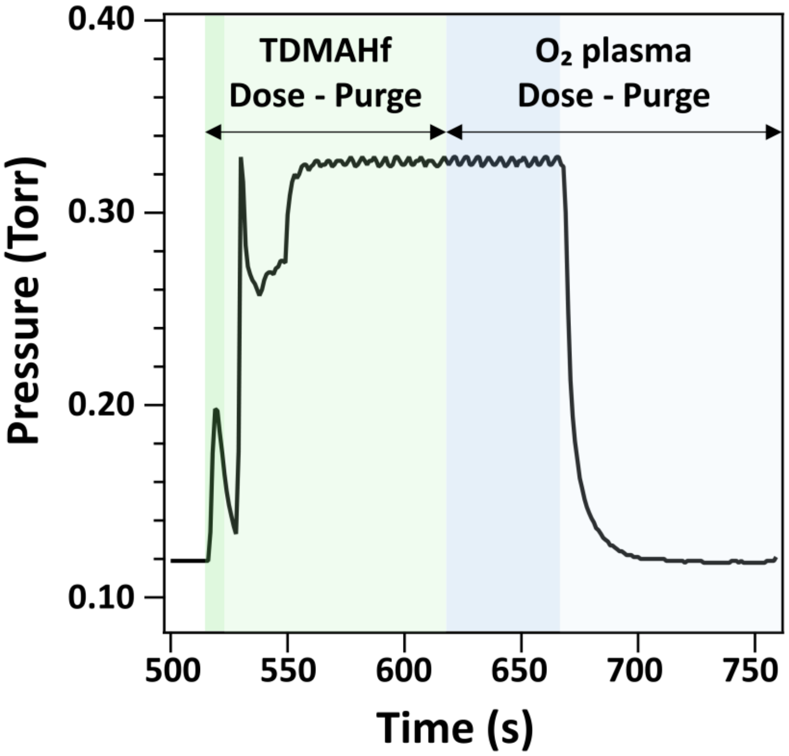

Figure S2. The pressure log of a supercycle consisting of TDMAHf half cycle [TDMAHf dose – purge] and O_2 plasma half cycle [O_2 plasma dose – purge].

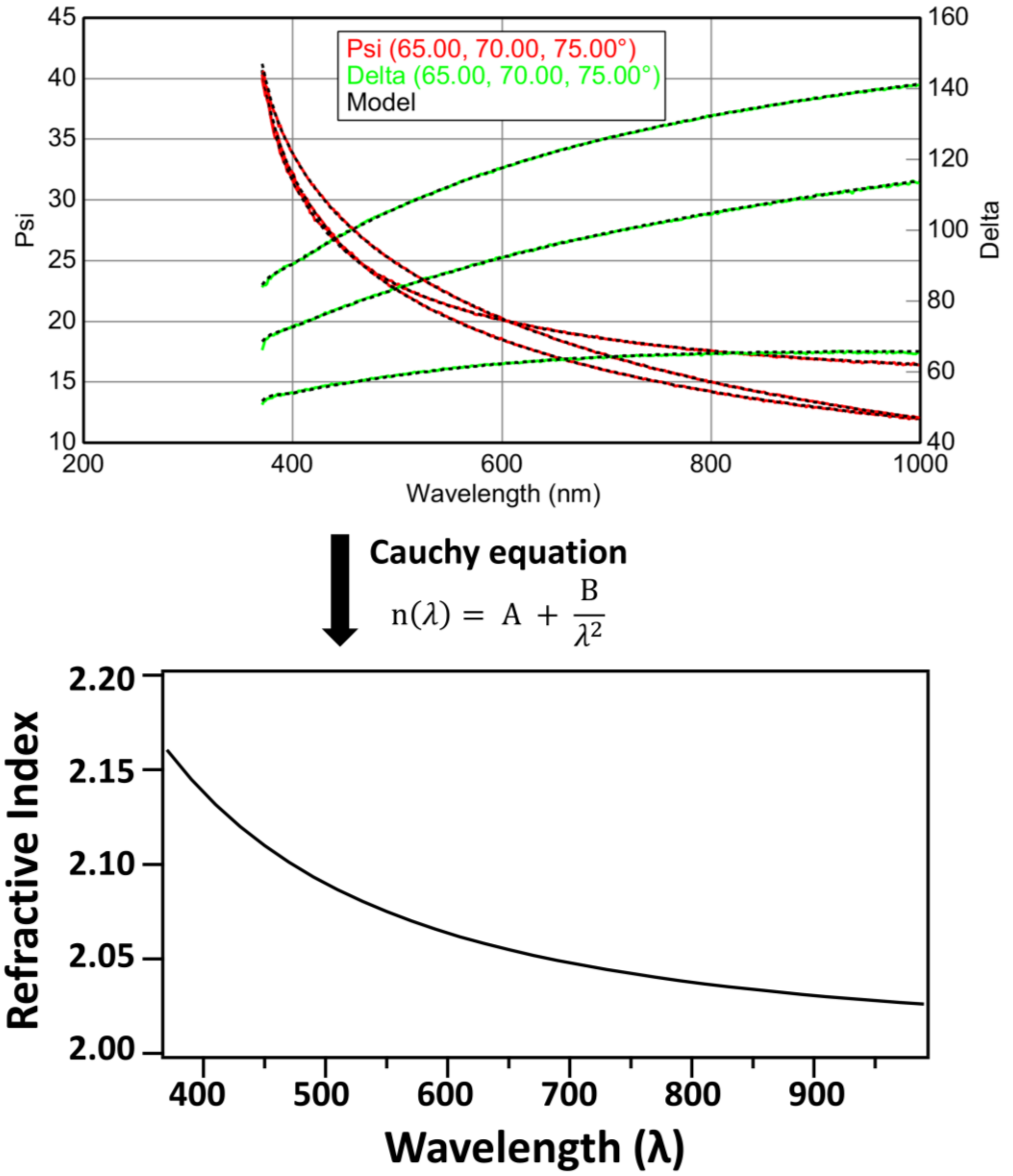

Figure S3. An example of SE data analyzed using Cauchy equation.

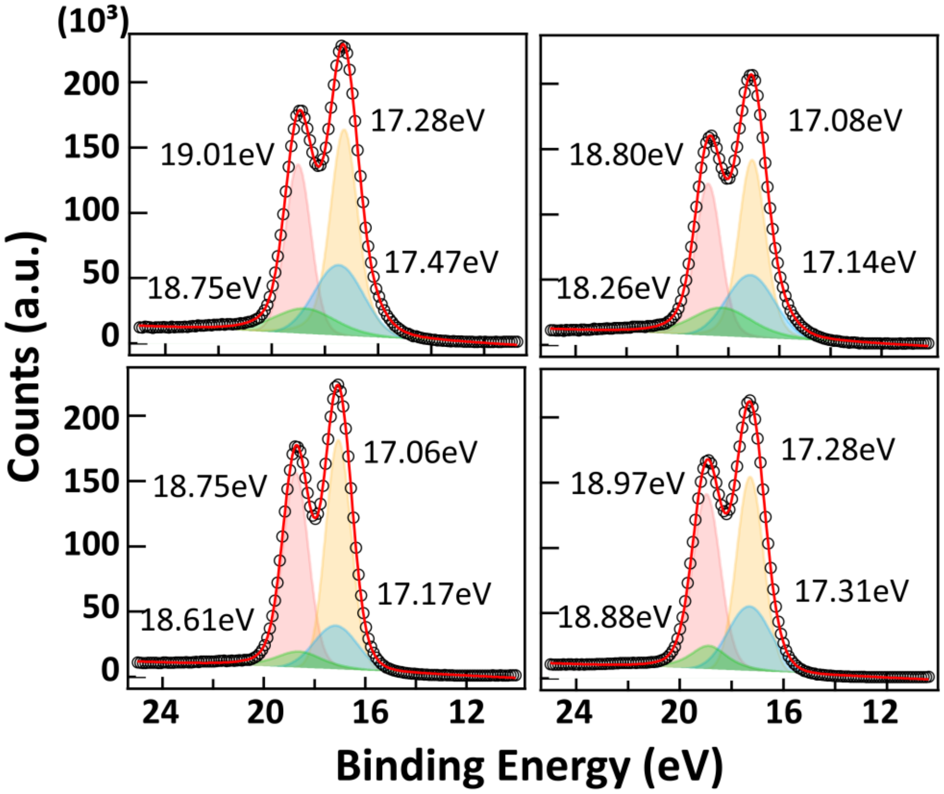

Figure S4. The Hf 4f HRXPS spectra deconvoluted into four peaks of the HfO_2 films deposited at different O_2 plasma conditions. (Top panel: O_2 flow rate 10 sccm, lower panel: O_2 flow rate 50 sccm, left panel: O_2 plasma power 20 W, right panel: O_2 plasma power 300 W.)
